# AAV-based gene delivery of antimicrobial peptides to combat drug-resistant pathogens

**DOI:** 10.1128/aem.01702-24

**Published:** 2025-01-06

**Authors:** Piyush Baindara, Dinata Roy, Chandra S. Boosani, Santi M. Mandal, Jonathan A. Green

**Affiliations:** 1 Animal Sciences Research Center, Division of Animal Sciences, University of Missouri 12271 , Columbia, Missouri, USA; 2 Department of Biological Sciences, Indian Institute of Science Education and Research Kolkata 99007 , Kolkata, India; 3 Department of Chemistry and Biochemistry, University of California San Diego 209674 , La Jolla, California, USA; Centers for Disease Control and Prevention, Atlanta, Georgia, USA

**Keywords:** antimicrobial peptides, adeno-associated virus, gene delivery, infectious disease, cancer

## Abstract

Antimicrobial peptides (AMPs) have emerged as potential alternatives to conventional antibiotics due to their novelty and multiple mechanisms of action. Because they are peptides, AMPs are amenable to bioengineering and suitable for cloning and expression at large production scales. However, the efficient delivery of AMPs is an unaddressed issue, particularly due to their large size, possible toxicities, and the development of adverse immune responses. Here, we have reviewed the possibilities of adeno-associated virus (AAV)-based localized gene delivery of AMPs for the treatment of infectious diseases with a special focus on respiratory infections. By discussing the gene delivery mechanism of AAV and the accompanying technical and therapeutic challenges with AMPs, we describe a foundation that emphasizes the use of viral vector-based gene delivery of AMPs for disease treatment.

## INTRODUCTION

Due to the rise of drug resistance toward traditional antibiotics, interest in alternatives to conventional antibiotics and anti-infectious therapies has increased (1). Consequently, the identification of advanced antimicrobial agents and the characterization of organisms that naturally produce such compounds are being explored. Antimicrobial peptides (AMPs) are a promising option that may serve as an alternative to traditional antibiotics because they may exhibit a decreased susceptibility to the development of microbial drug resistance. AMPs are broadly distributed across different genera. They range from simple prokaryotes to complex eukaryotes and represent as components of the host defense system (2). Indeed, AMPs isolated from different sources have been explored and are useful for inhibiting drug-resistant bacteria. Other studies have suggested a role in ameliorating clinical pathologies, such as cancer. Although efficient delivery of AMPs has always remained an issue, direct or localized gene delivery of AMPs has not been given much attention (3).

In humans and other mammals, the capacity to deliver foreign genes via gene therapy has shown great promise toward battling both genetic and acquired diseases. Viral vector-based gene delivery systems are a powerful tool that has been widely explored. The use of these delivery agents changed the face of conventional drug-based therapies for their effective therapeutic applications (4). Due to the demonstrable success in preclinical and clinical trials, the regulatory approval of viral vector-based therapies in treating different diseases has increased (4). Most viral vector-based gene therapies are used to address conditions arising from gene mutations, such as cystic fibrosis, sickle cell anemia, muscular dystrophy, etc.; however, the use of these therapies for the treatment of infectious diseases like influenza, malaria, and tuberculosis remains a challenge (5). Robust immuno-modulatory mechanisms and rapid mutation rates allow for the development of drug resistance in pathogens, and these aspects represent major roadblocks in the battle against infectious disease. Along with their high *in* vivo transduction efficiency and established clinical efficacy, adeno-associated viruses (AAVs) may provide stable transgene expression of AMPs upon single delivery. Especially, recombinant (r)AAVs driving expression of AMPs could offer a potential solution to fight against the current hurdles of pathogenic immune invasion and gene alterations, which give rise to drug resistance. Overall, this review summarizes the use of AAV-based gene delivery of AMPs as a potential avenue for combating infectious diseases. This review will also discuss the advantages and technical challenges arising from the use of AAVs to deliver AMPs.

## ADENO-ASSOCIATED VIRUS VECTORS

**Fig 1:**
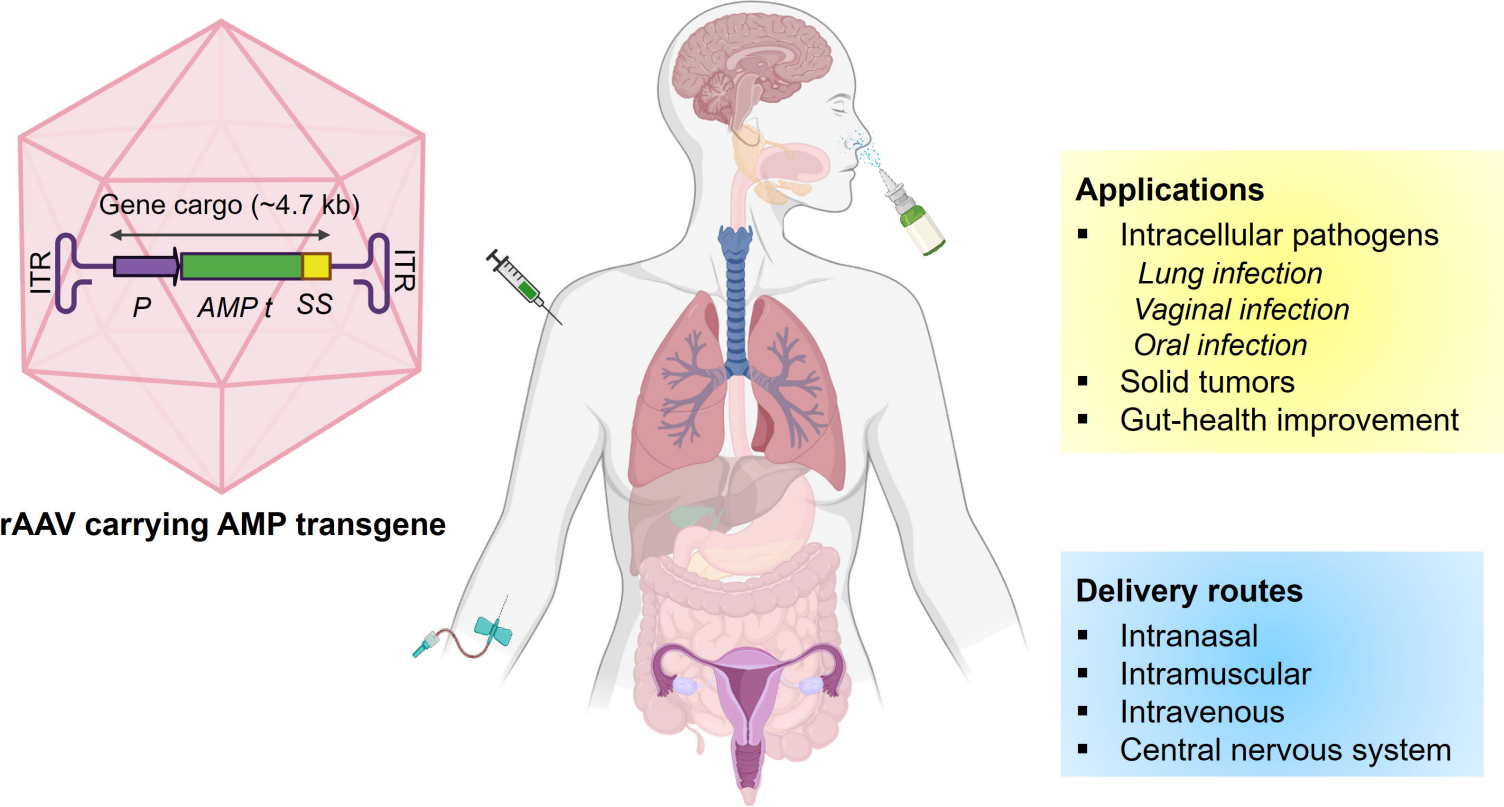
Applications and delivery routes of the AMP transgene carrying AAV. P: promoter; ITR: inverted terminal repeats; AMP t: AMP transgene; SS: signal sequence.

Adeno-associated viruses are one of the most extensively used gene therapy vehicles (4). AAV belong to the class of parvovirus that mainly requires co-infection with adenovirus to multiply. Several AAV serotypes were characterized from different species, and all contain a single-stranded DNA genome of around 4.8 kilobases with three genes, *Rep*, *Cap*, and *aap*. The three open reading frames are flanked by inverted terminal repeats (ITRs) and required for replication and packaging. The *Rep* gene ORF encodes four non-structural proteins (Rep78, Rep68, Rep52, and Rep40) involved in replication, transcriptional regulation, genomic integration, and virion assembly. The *Cap* gene ORF encodes three structural genes (VP1, VP2, and VP3) that are required for viral capsid formation. AAV capsid is an assembly of 60-mer repeated units forming an icosahedral structure that protects the viral genome as an outer capsid shell and also plays an essential role during cell binding and internalization (6). The assembly-activating protein (AAP) is encoded by the *aap* gene that overlaps with the *Cap* gene ORF. The AAP protein is required for nuclear localization and functional assembly of the capsid proteins (7). In the recombinant AAV (rAAV) employed for gene therapies, the gene of interest to be delivered and expressed in targeted cells is inserted between the ITRs replacing *Rep* and *Cap* (8). Ultimately, the rAAV functions as a protein-based nanoparticle containing the gene of interest. It can transduce both dividing and non-dividing cells by crossing the cell membrane while delivering the DNA cargo into the nucleus. As *Rep* is absent in rAAVs, the ITR-flanked transgenes are expressed, assembled into circular concatemers, and remain as episomes in the nucleus of transduced cells (9). The recombinant episomal DNA that contains the gene of interest does not integrate into the host genome and eventually becomes segregated with successive cell divisions. Furthermore, more than 12 naturally occurring serotypes and more than 100 variants of AAV have been tested and employed in various human gene therapy studies, targeting different tissues and organs (10). Also, AAV-based gene therapies have been employed with different routes of delivery, including intranasal, intravenous, and intramuscular, with reported efficient gene delivery and outcomes (Fig. 1) (11, 12). Overall, these qualities made rAAV a perfect candidate for gene therapies. Although rAAVs are widely used for gene delivery, self-complimentary AAVs (scAAVs) offer unique advantages, especially with the expression of the transgene as they favor higher and prolonged expression and better stability of the delivered DNA *in vivo*.

## RATIONALE FOR AAV-BASED GENE DELIVERY OF AMPs

The AMPs are present in a range of species, including prokaryotes and eukaryotes, and most are less than 50 aa in length. The short length of the AMP coding sequence lends itself to packaging into gene delivery agents, such as AAVs. AMPs that do not require any enzymatic posttranslational modifications are more suitable for AAV-based gene delivery due to favorable gene size for the delivery system (13). Interestingly, there are classes of AMPs that do not possess leader sequences and do not undergo posttranslational modifications for the production of the final active product, which may be particularly suitable for inclusion in AAV expression systems (14). Laterosporulins are another potential class of AMPs that do not possess leader sequences and do not undergo posttranslational modification other than disulfide bond formation, similar to human defensins (15, 16). Interestingly, laterosporulin10 has potent activity against the human pathogen, Mtb H37Rv. Most notably, it can kill the bacteria residing in intracellular organelles (15). This means that AMPs, such as laterosporulin10, could be easily delivered using an AAV vector to target intracellular pathogens while not needing any PTMs and transport after their gene expression within the cell. Notably, AAV-based AMP gene therapy might be limited to cytoplasmic-associated infections only until associated with an extracellular secretion system. Also, the extracellular secretion of expressed AMPs is not required when intracellular pathogens are the primary target in using AAV-based AMP gene delivery; however, the inclusion of specific signal sequences to the AMP’s transgene could provide a more focused strategy to target intracellular pathogens. Recombinant adenovirus carrying human *β*-defensin-3 (HBD-3) transgene has already been shown to prevent reactivation of latent tuberculosis in a mouse latent infection model (H37Rv) upon intratracheal delivery (17). This suggests the potential of viral vector-based gene delivery, such as AAVs, as a suitable means to deliver AMPs in fighting against infectious diseases. Similarly, adenovirus-based gene delivery of LL-37, HBD-2, and HBD-3 was found successful in reducing the bacterial load in diabetic and burn wound infection models (18–20). Also, the AAV8-based intravaginal delivery of HBD-3 was demonstrated to efficiently clear *Escherichia coli* infection in the uterine cavity of mice, rescuing the animals from ascending infection-related preterm birth (21). Conclusively, it seems that AMPs could be potentially used in AAV-based therapies to fight against infectious diseases. Interestingly, AMPs of bacterial origin have not been explored for their feasibility using AAV-based gene delivery strategies to treat infectious diseases. Indeed, AMPs have an enormous capacity to prevent microbial infections (22). Consequently, they may serve as another tool to augment conventional antibiotics or even act as effective alternatives to antibiotics in an effort to limit the rise of microbial drug resistance (23).

The strategy of intranasal and inhaled pulmonary delivery of aerosolized AAV is successful in gene delivery to the lungs, as reported in influenza and cystic fibrosis (24–26). The AAV9-based delivery and expression of a broadly neutralizing antibody F16 are reported to provide efficient protection against influenza A upon intranasal delivery (24). Another study showed that a single intramuscular dose of (r)AAV vector expressing H1 hemagglutinin (HA) elicits strong neutralizing and broadly protective antibodies against influenza (27). Next, the (r)AAV-based intranasal delivery of the CF transmembrane regulator (*CFTR*) gene in human patients directs the efficient use of AAV-based gene therapies for cystic fibrosis (28). Another clinical trial has also been conducted using a synthetic AAV vector (A101) and a human *CFTR* gene consisting of a deletion in its regulatory domain (*CFTRΔR*) (29). These experimental studies suggest that the AAV-based gene delivery of AMPs could also be employed for localized lung delivery to treat infectious respiratory diseases. Additionally, AMPs are also effective against respiratory pathogens, including severe acute respiratory syndrome coronavirus 2 (SARS-CoV-2), where gene delivery of AMPs might open new avenues to treat infectious diseases (15, 30, 31). Interestingly, the AAV-based gene delivery to combat SARS-CoV-2 is reported promising in different studies by targeting or delivering spike gene, anti-human angiotensin-converting enzyme 2 (hACE2) antibody (ch2H2), ACE2 decoy protein, and short hairpin RNAs against RdRp and N gene via intranasal, intratracheal, or intramuscular injection; however, the direct gene delivery of an anti-SARS-CoV-2 AMP could be a new strategy (32–35). Furthermore, the lung-specific gene delivery of AMPs with AAVs has the advantage of restricting the expression of AMPs to respiratory epithelia and avoids dissemination throughout the body. More advantageously, the natural turnover rate of respiratory epithelial cells warrants subsequent and successful elimination of AAVs from the body in a certain time frame that addresses the issues arising from long-term viral exposure in the host (5, 36). In addition to direct lung delivery, oral administration of AAV containing the AMP gene is another attractive delivery option that is non-invasive and potentially transduces the whole gut epithelia (37). In this scenario, the immunomodulatory and anti-inflammatory properties of AMPs may be beneficial in treating gut infections or gut inflammatory disorders (38, 39).

Additionally, AMPs are reported to have potential anticancer properties that they can exert through various mechanisms, as found in both *in vitro* and *in vivo* animal model studies (40, 41). Consequently, tissue-specific localized delivery of AMPs by AAVs could serve as an anticancer strategy and may be particularly useful when applied with minimally invasive delivery methods, such as endoscopy and tissue ablation. Direct delivery of AAVs, such as in the case of lung and colon cancer treatment, is one of the examples that can be investigated. Furthermore, conventional treatment strategies using AMPs have major limitations in terms of huge quantity, costs, production efficiencies, and immunogenic responses. Despite this, the AAV-based localized delivery of AMPs seems to overcome the associated limitations with conventional treatment methods, especially in the case of infections caused by intracellular pathogens and in cancer therapeutics. Interestingly, the AAV-based delivery of AMPs appears to be effective, where AMP containing AAV infects the cells and initiates expression and secretion of AMPs that subsequently kill the intracellularly residing pathogens. The sections that follow will discuss the advantages, challenges, and specific strategies that can be employed and shed light on the potential and applications of the AAV-mediated AMP delivery.

## ADVANTAGES OF AAV-BASED GENE DELIVERY

Other than being associated with some disadvantages that were discussed above, AAV remains the most widely used and tested delivery method in preclinical and animal model studies. One reason for this is the stable long-term transgene expression in different cell types of targeted tissues and organs (42). AAV vector-based gene therapies have also exhibited safety and efficacy in various clinical and preclinical trials. Some of them have already been approved for use in humans (4, 43). Although AAVs were first identified as coinfected viruses along with adenovirus, they can independently infect cells of humans and other mammals. However, the fact that AAV infections in humans are not life-threatening or are not known to cause any serious illness in healthy individuals aligns with the safe use of AAVs in human therapeutics (44). An interesting fact is that more than 90% of the human population was found to be seropositive for different serotypes of AAV, yet it did not present any adverse immune response. However, the recurrent delivery of rAAV vectors containing specific transgenes has the potential to elicit an immune response (45, 46). When different AAV serotypes were administered, each serotype may initiate an immune response differently; however, results from several clinical trials suggest that rAAV vectors do not prompt a specific immune response that can impede treatment efforts (47). Additionally, as AAVs are not known to cause any human disease, they are categorized under lower biosafety levels and are generally handled or managed at BSL-1 levels, which facilitates AAV vector-based manipulations, including *in vivo* delivery or treatment experiments.

## CHALLENGES WITH AAV-BASED GENE DELIVERY

The increasing number of rAAV-based clinical trials employing various gene therapies suggests that rAAV-based gene therapies have great potential. Even so, their use still faces several obstacles that need to be overcome. As in the case of any gene therapy, immunogenicity is one of the biggest challenges, and this is true in the case of rAAV-based gene therapies, as well. Immune responses in the host arising from the generation of neutralizing antibodies (NAbs) against the foreign transgene and viral capsid proteins are ways that rAAV-based gene therapies can become compromised. Finding a suitable novel capsid or capsid engineering is one strategy to address NAbs-related issues (48). In the case where customization of the vector platform is not possible for the individual patient, plasmapheresis is one option to remove anti-AAV NAbs from the bloodstream. Another option is to treat with IgG-cleaving endopeptidases, such as imlifidase, to non-specifically lower the IgG Abs from patient sera (49). The serotype-specific immune response can also be managed easily as reported in the case of AAV2 and AAV8 where the preferred receptor is hairpin proteoglycans for AAV2 that may elicit a greater T-cell response (50). Furthermore, adaptive immunity against rAAV can be evaded by targeting unprofessional APCs, such as dendritic cells, by driving tissue-specific expression, which can be controlled by designing miRNA binding sites for miR142 that can halt transgene expression (51).

Growing evidence suggests that in addition to adaptive immune response, the rAAV vector genome may also trigger innate immune responses, such as those arising from the methylation-free CpG inserts that can be detected by the TLR9 pathway (52). Other mechanisms involving RIG-I (*Ddx58*), CGAS (*Mb21d1*), and STING (*Tmem173*) may also play a role in the detection of foreign DNA (53). Ultimately, the major challenge that needs to be addressed would be determining the appropriate dosages for treatment, which could avoid robust immune responses and consequent toxicities, as has been observed in recent rAAV-based gene therapies (54). Additionally, preexisting immunity to AAV vectors discussed earlier is another concern along with random integration into the host genome that might lead to off-target effects that affect the endogenous expression of transgene via unintended activation or inhibition (55). On this note, integration of the gene sequences from the AAV-mediated delivery at the AAVS1 locus appears to be a safe harbor for transgene integration. Another important aspect is that the expression patterns of different AAV serotypes are unpredictable among different tissues or organs, which might be due to variable efficacies in cellular entry (56). More than 12 naturally occurring serotypes of AAV were tested and reported to be differentially efficient, as was seen in various human gene therapies targeting different tissues and organs (10). In the current context, even though we have achieved remarkable progress in AAV-based gene therapies, further investigation is required to develop strategies for better capsids, delivery routes, and vector genome design. Specific delivery strategies should be developed for the particular infectious disease condition to achieve the efficient output of gene therapy.

## FACTS AND CONSIDERATIONS FOR AAV-BASED DELIVERY OF AMPs

1. AMPs encoded by a single gene (<5 kb) with no requirement of posttranslational modifications for the final active product are most suitable for the AAV-based gene delivery.
2. Technically, in the case of intracellular pathogens or cancers, extracellular secretion or transportation of AMP is not necessary, making AMP sequences an ideal delivery candidate.
3. AMPs are known to have diverse bioactivities, including anti-cancer, anti-bacterial, and anti-viral properties; thus, AMPs may also possess anti-AAV activity and can destroy the AAV itself (57).
4. AAV allows *de novo* synthesis of foreign peptides with the required conformation and topology, which is a plus for AMPs.
5. Localized delivery of AMPs can be a preferred option to avoid any adverse immune response or toxicities. AAV’s stability and wide tropism toward various cell types and tissues make them perfect vehicles for AMP applications.
6. The respiratory tract is well coated with surfactants; thus, prior treatment might be required for efficient delivery of AAV-carrying AMPs. This is applicable in the case of respiratory, rectal, or oral administration of AAVs.
7. A suitable animal model resembling human infectious disease and cancer in terms of immune response or pathogenesis needs to be predetermined for the AAV-based gene delivery of AMPs.
8. Understanding the pathogenesis of targeted infection disease and pathogens along with AMP mechanisms of action against the pathogen is essentially required to address important questions on the desired effects of transgene and the status of disease in targeted cells or tissues.
9. The turnover rate of the targeted cells is also important, along with the AMP degradation rate in the biological system, to determine or estimate the required dosing.
10. Finally, better-designed preclinical trials are required to explore the potential of the AAV-based gene delivery of AMPs.

## CONCLUDING REMARKS

Although the AAV-based gene delivery of AMP needs further investigation and optimization, it provides therapeutic opportunities to combat infectious diseases and cancer. At the University of Missouri, Columbia, we conduct *in* vivo gene editing in pigs using AAVs targeting different organs inculding the brain and lungs. Based on our expertise here we discussed the benefits and considerations for AAV-based gene delivery of AMPs. There are several AMPs known that can be tested using the AAV-based gene delivery platform to explore their potential against several infectious pathogens and tumors. Specifically, bacterial diversity is enormous and possesses a huge potential to have several novel AMPs that can avail endless opportunities to fight drug resistance in infectious diseases and cancer. Given the antimicrobial and anticancer potential of AMPs along with other immunomodulatory properties, the discussed strategies suggested potential approaches to fight against drug-resistant intracellular pathogens or tumors.
